# Cleft palate in fetuses: feasibility of early diagnosis by Crystal and Realistic Vue rendering 3D ultrasound technology in the first trimester

**DOI:** 10.3389/fped.2023.1199965

**Published:** 2023-07-14

**Authors:** Zhihong Shi, Huaxuan Wen, Junhong Leng, Junjun Wang, Yuemei Wang, Dandan Luo, Zhixuan Chen, Yue Qin, Meiling Liang, Ying Tan, Shengli Li

**Affiliations:** ^1^ Department of Ultrasound, Shenzhen Maternity and Child Healthcare Hospital, Shandong University, Shenzhen, China; ^2^ Department of Ultrasound, Jinan Maternity and Child Care Hospital, Jinan, China; ^3^ Department of Ultrasound, Affiliated Shenzhen Maternity and Child Healthcare Hospital, Southern Medical University, Shenzhen, China

**Keywords:** cleft palate, fetal 3D ultrasound, Crystal and Realistic Vue technique, first trimester, early diagnosis

## Abstract

**Objectives:**

This study aimed to evaluate the feasibility of direct visualization of a normal fetal palate and detect cleft palate in the first trimester with a novel three-dimensional ultrasound (3D US) technique, Crystal and Realistic Vue (CRV) rendering technology.

**Methods:**

Two-dimensional (2D) images and 3D volumes of healthy and cleft palate fetuses at 11–13^+6^ weeks were obtained prospectively. 2D ultrasound views included the coronal view of the retronasal triangle and the midsagittal view of the face. 3D-CRV views were analyzed by multiplanar mode display. The pregnancy outcomes of all fetuses were determined during the follow-up period.

**Results:**

In our study, 124 fetuses were recruited, including 100 healthy fetuses and 24 cleft palate fetuses. The cleft palate with lip was observed in 23 fetuses (bilateral in 15, unilateral in 6, median in 2), and one cleft palate was only found in the abnormal group. The bilateral (*n* = 12) and median (*n* = 2) cleft palates with lips and the cleft palate alone (*n* = 1) were associated with other anatomical or chromosomal abnormalities, and one unilateral cleft palate with cleft lip had concomitant NT thickening. In the cleft palate fetus group, 16 fetuses suffered intrauterine death, which was associated with other structural or chromosomal abnormalities in 14 fetuses, seven cases were terminated after consultation, and one was delivered at term. The coronal view of the retronasal triangle and the midsagittal view was easily obtained in all fetuses. 3D-CRV images of palatal parts were clearly obtained in all cases. Unilateral, bilateral, and median cleft palates with cleft lips were visually demonstrated and classified by the 3D-CRV technique.

**Conclusion:**

It is feasible to identify the palate by 3D-CRV in the first trimester in both healthy and cleft palate fetuses. Together with 2D ultrasonography as a complementary diagnostic tool, 3D-CRV is helpful in classifying the cleft palate with a reasonable degree of certainty.

## 1. Introduction

The fetal hard palate is composed of a primary palate and a secondary palate. The primary palate is only the front small part of the hard palate, which includes the alveolar bone of four incisor teeth. The secondary palate is formed from paired palatal shelves that are outgrowths of the maxillary processes. By the tenth week, the primary palate and nasal septum have fused with the secondary palate. The failure in palate formation may lead to congenital developmental abnormalities such as cleft palate (1). The risk factors include paternal age, previous cases in family members, smoking habits, and exposure to some medication or home products. A comprehensive systematic review and meta-analysis reported the prevalence of cleft palate was 0.33/1,000, that of cleft lip was 0.3/1,000, and that of cleft lip and palate was 0.45/1,000 (2). The fetal cleft palate with or without a cleft lip is often associated with other anatomical or chromosomal abnormalities and with more than 500 syndromes (3–5). Jean et al. investigated 43 fetuses with cleft palate but without cleft lip, and the results showed it was associated with other malformations in 34 fetuses (6). This result was also confirmed by the multicenter study of Clementi et al. (7) and the prospective study of Offerdal et al. (8). Therefore, in a high-risk group, the diagnosis of cleft palate may contribute to improving the prenatal detection of fetal anomalies, especially those associated with other anatomical or chromosomal abnormalities.

A facial cleft is mainly screened antenatally by visualizing the upper lip on routine scanning in the mid-trimester (9). It is not mandatory to assess the palate of the fetuses in the uterus, and therefore the detection rate of cleft palate is very low. Approximately 0%–22% of cleft palate is identified antenatally in the gestation period (10). With the progression of prenatal diagnosis, the detection of cleft palate has gradually attracted the attention of prenatal sonographers. Reports about visualizing fetal hard palate are still limited. Florent et al. reported an easy method for assessing fetal hard palate by two-dimensional (2D) ultrasonography: the axial transverse view (ATV) (11). They also proposed a simple image scoring system, which was easy and helpful for sonographers to accurately identify the palate structure (12). However, the assessment of the palate is still a challenge due to the craniofacial bone shadow in the second and third trimesters. In recent years, multiple signs in the first trimester have been reported for the detection of cleft palate. The coronal view of the retronasal triangle (RNT) was suggested as a useful tool to assess the primary palate in the first trimester (13). Lakshmy et al. reported using the “superimposed-line” sign (SLS) in the early diagnosis of the cleft of the secondary palate on 2D imaging of the vomeromaxillary junction in the midsagittal view (14).

3D techniques have also been introduced for the detection of palatine clefts in early pregnancy (15, 16). In the study of Martinez-Ten et al., the primary palate and secondary palate were visualized adequately by 3D ultrasonography in the first trimester (17). Crystal Vue (CV) is a rendering technology based on image-contrast enhancement and has proven to be potentially effective in differentiating between soft tissue and bony structure (18). The technique has been used for the observation of the fetal central nervous system (19), spine (20), esophagus (21), and placenta accreta spectrum (22). Lees et al. qualitatively evaluated the performance of this novel tool in the assessment of the face of healthy fetuses and those with cleft lip and palate in the second trimester (23). However, it is difficult to obtain high-quality 3D ultrasound volume and it takes a long time in the second and third trimesters (24, 25). In this study, the novel 3D technique, Crystal and Realistic Vue (CRV) rendering technology, was employed to visualize fetal integrally hard palate, which may be helpful for the diagnosis of cleft palate in the first trimester.

## 2. Methods

The fetal orofacial 2D images and 3D volumes were collected from healthy and cleft palate fetuses at the Department of Ultrasound in a tertiary-care maternity and children medical center, from January 2021 to December 2021. This study was approved by the Ethics Committee of the hospital, and informed consent was obtained from all pregnant women. The outcomes were obtained from the Nation Birth Defects Monitoring Network, maternity medical records, and phone calls. The lip, alveolar bone, and palate structures were recorded.

There are two groups of fetuses in this study: healthy fetuses and cleft palate fetuses. The characteristics of the healthy fetus group are as follows: (1) singleton pregnancy was at 11–13^+6^ weeks; (2) gestational age (GA) based on the crown-lump length (CRL) was consistent with the last menstrual period (LMP); (3) the facial structure of the fetus was normal (we evaluated the fetal face in the first trimester by 2D ultrasonography of the coronal view of RNT and the midsagittal view of the face; the normal facial structure was defined based on the continuity of the base of RNT and maxillary gap invisible or <1.5 mm); (4) the combined test for aneuploidy had low risk in the first trimester; and (5) high-quality 2D ultrasound images and 3D ultrasound volumes could be obtained. The characteristics of the cleft palate fetus group are as follows: (1) fetuses with or without cleft palate were suspected at 11–13^+6^ weeks, and (2) high-quality 2D ultrasound image and 3D ultrasound volume could be obtained. The fetus was excluded if the follow-up results were inconsistent with the findings from ultrasonography in the first trimester. If the fetus had a high risk for aneuploidy on the combined test or structural defects were present, fetal karyotyping or the chromosomal microarray assay by chorionic villus sampling or amniocentesis was recommended.

Using Samsung W10 and WS80A ultrasound systems equipped with a CV4–8 MHz transabdominal volumetric probe (Samsung Medison Co. Ltd., Seoul, South Korea), 2D US views and 3D US volumes were collected by two experienced ultrasound specialists. 2D ultrasound views required for cleft palate screening are as follows: the coronal view of RNT and the midsagittal view of the face. The initial view of 3D ultrasound volume scanning was the midsagittal section of the fetal face. After the midsagittal section of the fetal face was obtained, the probe was adjusted appropriately to make the ultrasound beam as perpendicular to the hard palate as possible. The quality of 3D-CRV images depends on high-quality 2D views. Hence, it is important to do prescanning first by swinging the probe in the scanning area until the 2D image was clear. Then, the size of the volume box was adjusted, in which the proportion of the fetal face is approximately three-fourths. The surface mode was used, and the quality of volume acquisition was set at “extreme”. The angle of the volume sweep was 50–60°. If the fetus moved during the scanning, the volume was deleted and then collected again. The volume data were stored for postprocessing.

**Figure 1:**
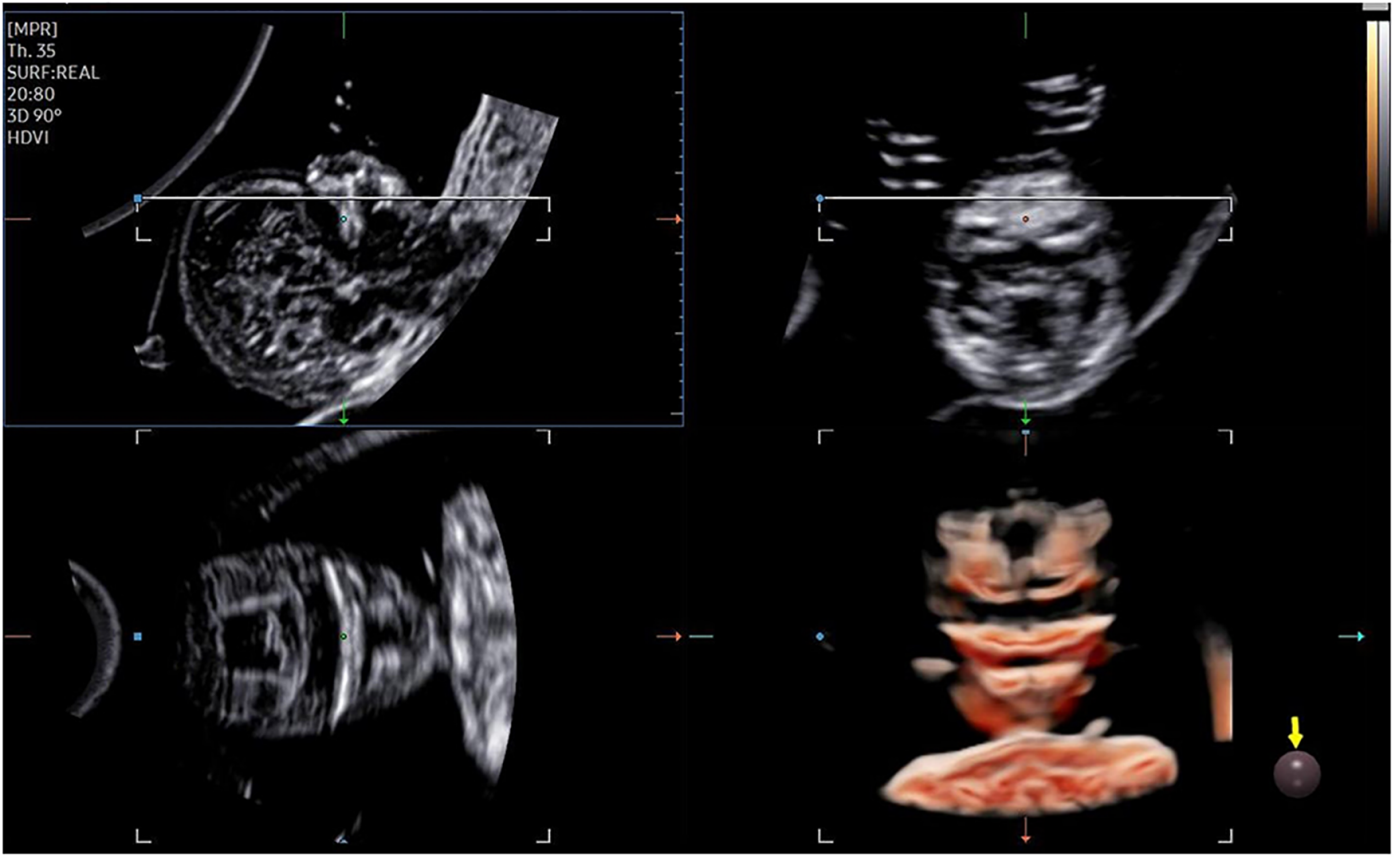
3D-CRV postprocessing: coronal section.

**Figure 2:**
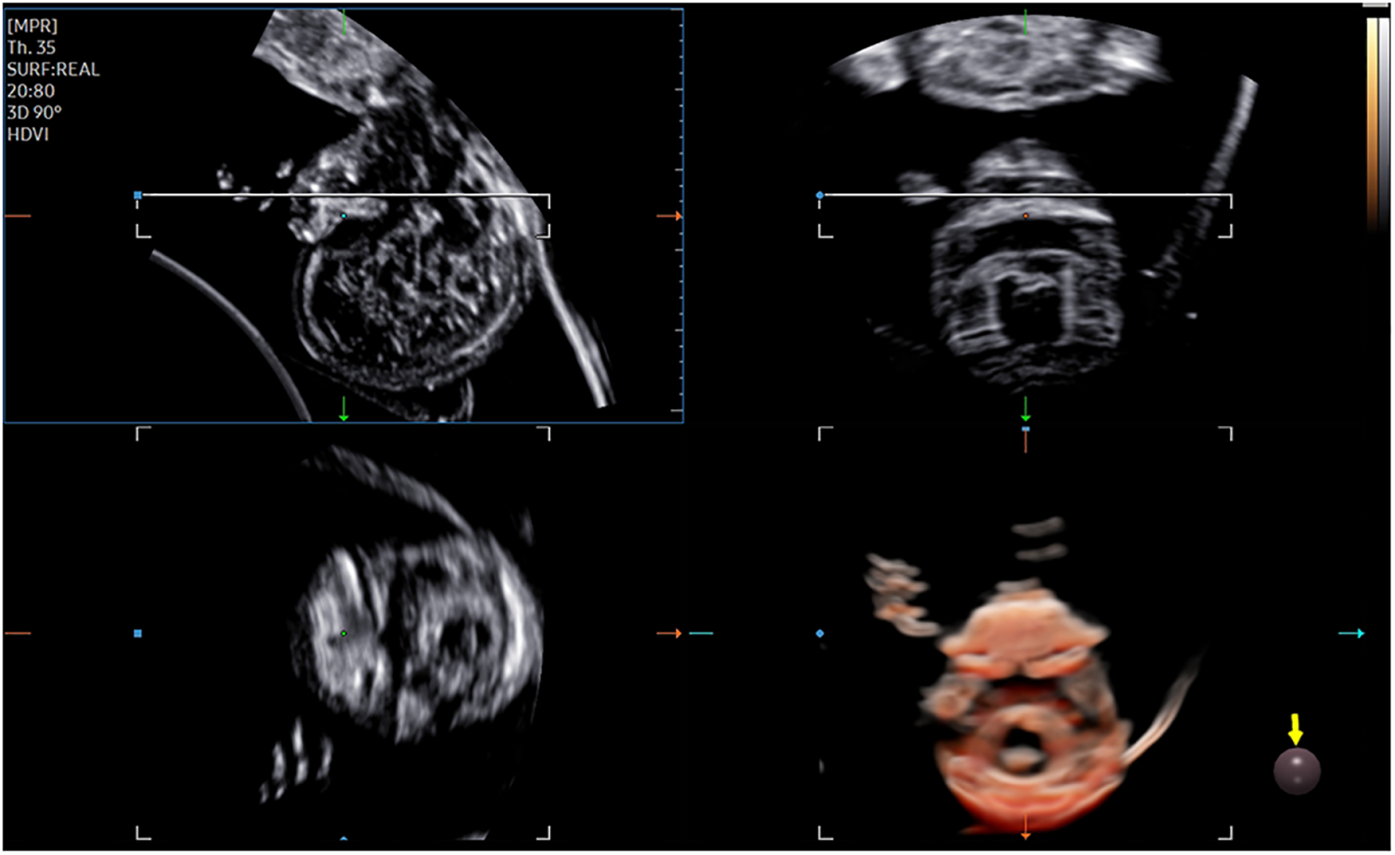
3D-CRV postprocessing: transverse section.

**Figure 3:**
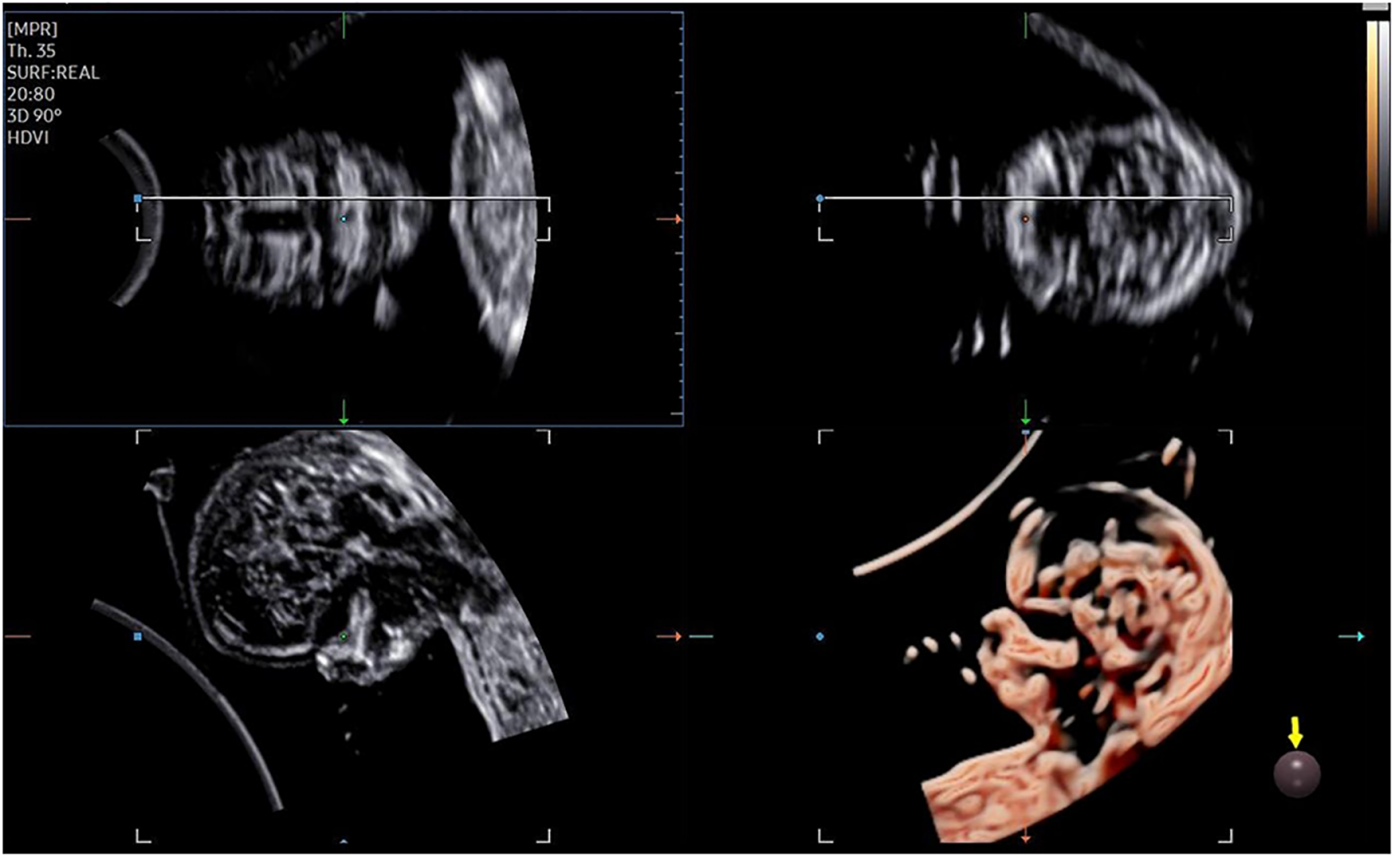
3D-CRV postprocessing: sagittal section.

The 3D-CRV rendering technology was used for postprocessing. To orient the volume and identify the primary palate and secondary palate, the multiplanar mode was applied. The sagittal view is shown in image A, and the transverse plane is shown in image B. The *X*, *Y*, and *Z* controls were used to adjust section A to obtain a midsagittal section of the face in section A. The region-of-interest (ROI) box was altered to decrease the size to maximize the resolution of the rendered image. By gradually moving the rendered image from back to front in section A (Figure 1), 3D-CRV coronal views of the primary and secondary palate were observed in sequence. Furthermore, the *Z* control was rotated in section A, so that the fetal head was downward and the hard palate was parallel to the border of the ROI box. When the palate was completely placed into the ROI box (Figure 2), the integrally hard palate was seen in one view. Finally, sections A and B were adjusted to obtain fetal profile images (Figure 3). Sagittal views of the hard palate were seen by fluctuating the rendered image. Notably, correctly changing the optimal gain, transparency, light source, and boundaries of the ROI box during these manipulations could display the bone better. When observing the soft tissues of the upper lip, transparency should be reduced as much as possible, but it should be increased appropriately when observing bony structure.

## 3. Results

A total of 124 fetuses were enrolled in the present study, including 24 fetuses with cleft palate and 100 healthy fetuses. The facial structure was normal in healthy fetuses on anatomical examination in the second trimester and postnatal follow-up. The findings from the follow-up of 24 fetuses with cleft palate were consistent with those from early pregnancy examinations. The median maternal age was 31 years (range: 23–40 years) in the healthy group and 33 years (range: 22–44 years) in the cleft palate group. The median GA at diagnosis was 12 weeks (range: 11^+3^–13^+4^ weeks) in the healthy group and 12^+4^ weeks (range: 11^+5^–13^+6^ weeks) in the cleft palate group. The mean CRL was 60.92 ± 6.39 mm in the healthy group and 60.98 ± 5.25 mm in the cleft palate group. The mean nuchal translucency (NT) value was 1.55 ± 0.35 mm in the healthy group and 2.62 ± 2.26 in the cleft palate group. Among these fetuses, cleft palate with lip was observed in 23 fetuses (bilateral in 15, unilateral in 6, median in 2) and one fetus had cleft palate alone in the cleft palate group. The characteristics of fetuses are summarized in Table 1.

**Table 1:** Characteristics of the cleft palate group.

Case no.	GA at diagnosis (weeks)	CRL (mm)	NT (mm)	Diagnosis	Associated anomalies	Chromosomal analysis	Pregnancy outcome
1	11^+5^	53	5.0	Cleft palate only	Skin edema; nasal bone dysplasia; omphalocele	Normal	Intrauterine death at 12^+4^ weeks
2	12^+3^	73.5	1.3	Unilateral CPL	–	Normal	Intrauterine death at 17^+5^ weeks
3	12	63.3	1.3	Unilateral CPL	–	–	Voluntary termination of pregnancy at 22 weeks
4	12^6^	60.7	1.0	Bilateral CPL	–	–	Voluntary termination of pregnancy at 13^+2^ weeks
5	12	54.1	1.1	Unilateral CPL	–	–	Intrauterine death at 15^+6^ weeks
6	13^+1^	67.8	2.7	Unilateral CPL	–	–	Intrauterine death at 14^+1^ weeks
7	12^+2^	58.2	1.1	Bilateral CPL	TOF; OSB; omphalocele; absence of radius	–	Intrauterine death at 13^+4^ weeks
8	13^+6^	67.5	2.8	Bilateral CPL	Cardiac abnormalities	–	Voluntary termination of pregnancy at 14^+6^ weeks
9	13	52.6	5.8	Bilateral CPL	Holoprosencephaly; cardiac abnormalities; omphalocele; skin edema; polydactyly	–	Intrauterine death at 17^+2^ weeks
10	12^+2^	62.1	2.2	Median CPL	Holoprosencephaly	Loss Xq25 1.3Mb	Intrauterine death at 13^+2^ weeks
11	13^+3^	61.2	1.5	Bilateral CPL	Absence of radius	–	Intrauterine death at 14^+3^ weeks
12	12^+4^	61.6	2.9	Bilateral CPL	Encephalomeningocele; omphalocele; amniotic band syndrome	–	Intrauterine death at 13^+4^ weeks
13	12^+6^	56.6	2.2	Bilateral CPL	Holoprosencephaly	–	Intrauterine death at 13^+5^ weeks
14	12^+4^	62.4	1.7	Bilateral CPL	–	Trisomy 13	Intrauterine death at 13^+2^ weeks
15	13^+6^	67.0	2.0	Bilateral CPL	Limb wall syndrome	–	Intrauterine death at 15^+4^ weeks
16	12^+4^	60.8	5.8	Bilateral CPL	Cardiac abnormalities	Trisomy 18	Intrauterine death at 13^+1^ weeks
17	12^+2^	58.0	1.6	Unilateral CPL	–	Normal	Delivery at 38 weeks
18	12^+2^	62.9	1.3	Bilateral CPL	–	−21/18	Voluntary termination of pregnancy at 15^+4^ weeks
19	13	62.8	1.1	Unilateral CPL	–	–	Voluntary termination of pregnancy at 14^+6^ weeks
20	13^+3^	59.5	1.5	Bilateral CPL	Holoprosencephaly	–	Intrauterine death at 17^+3^ weeks
21	12^+1^	56.1	1.3	Bilateral CPL	–	–	Voluntary termination of pregnancy at 13^+2^ weeks
22	12^+3^	68.4	1.7	Bilateral CPL	–	–	Voluntary termination of pregnancy at 16^+4^ weeks
23	12	57.0	8.4	Bilateral CPL	Cardiac abnormalities; skin edema	Trisomy 13	Intrauterine death at 12^+6^ weeks
24	12^+6^	56.3	7.8	Median CPL	Holoprosencephaly; cardiac abnormalities; skin edema	Trisomy 13	Intrauterine death at 13^+3^ weeks

GA, gestational age; CRL, crown-rump length; NT, nuchal translucency; CPL, cleft palate and lip; TOF, tetralogy of Fallot; OSB, opened spina bifida; –, without associated anomalies or chromosomal analysis.

In our study, fetuses in the cleft palate group had a high rate of other concomitant structural or chromosomal abnormalities and had a relatively poor prognosis. Only one fetus with unilateral cleft palate and lip was delivered at term (case 17), and karyotype analysis and the chromosomal microarray assay were normal. Eight fetuses with cleft palate and lip underwent genetic testing, five of whom had chromosomal abnormalities. Case 3 had a known history of cleft palate and lip during the pregnancy, but further genetic tests were not conducted. Of 24, 8 (33.3%) fetuses showed increased NT, while 15 (62.5%) (bilateral in 12, median in 2, and cleft palate alone in 1) showed other anatomical or chromosomal abnormalities. In addition, 16 fetuses suffered intrauterine death, which was associated with other structural or chromosomal abnormalities in 14 fetuses. The pregnancy was terminated after consultation in 7 cases, 5 of them had bilateral cleft palate and lip, and 1 of 5 had concomitant NT thickening. Unfortunately, the pregnancy was also terminated in the other two fetuses with unilateral cleft palate and lip.

**Figure 4:**
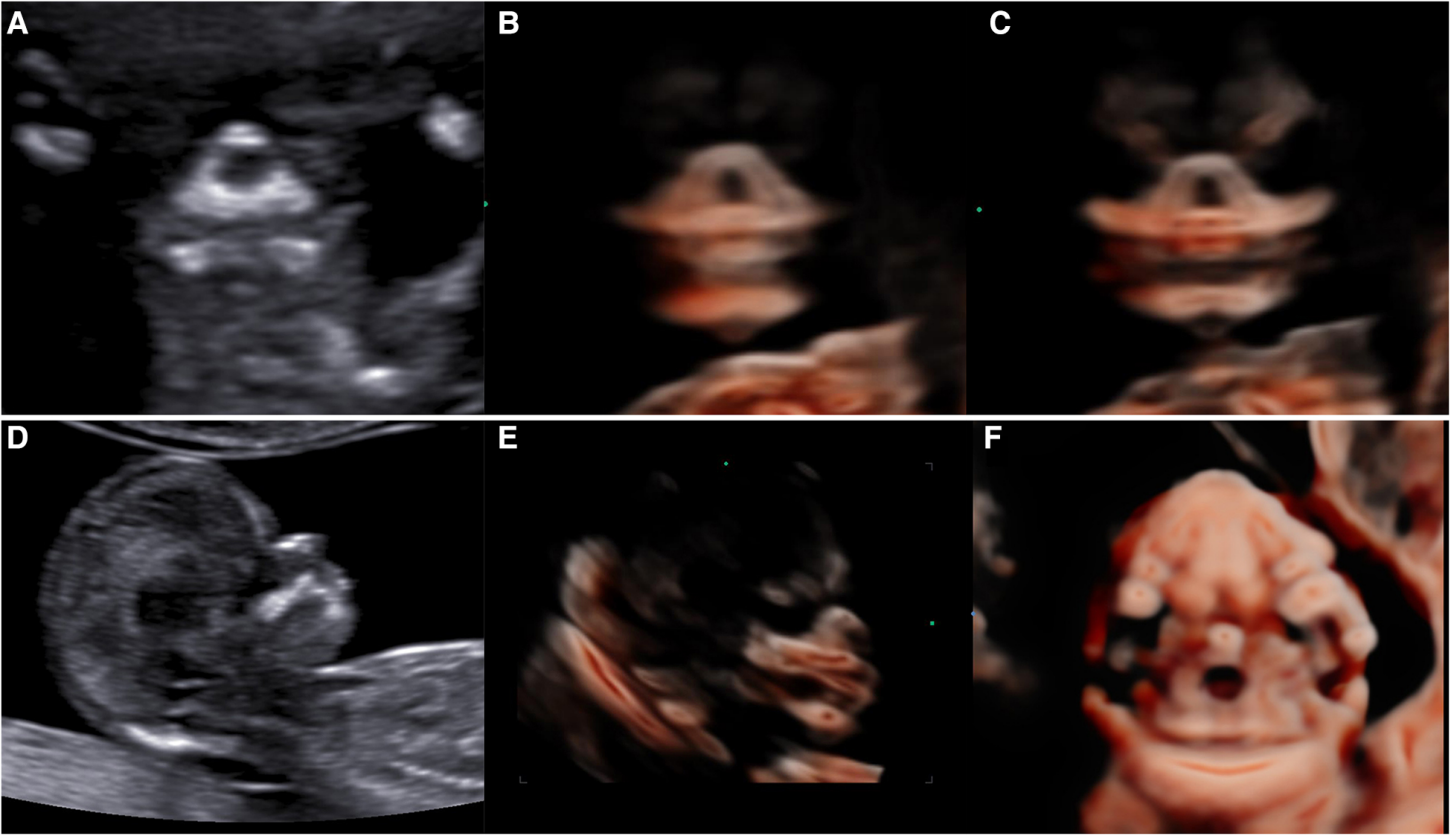
Normal 2D and 3D-CRV views. (**A,D**) Coronal view of RNT and the midsagittal view of a healthy fetus, respectively. (**B,C**) Series of 3D-CRV coronal views: the primary palate is shown in (**B**), and the secondary palate in (**C**).(**E**) 3D-CRV sagittal view. (**F**) 3D-CRV axial view.

**Figure 5:**
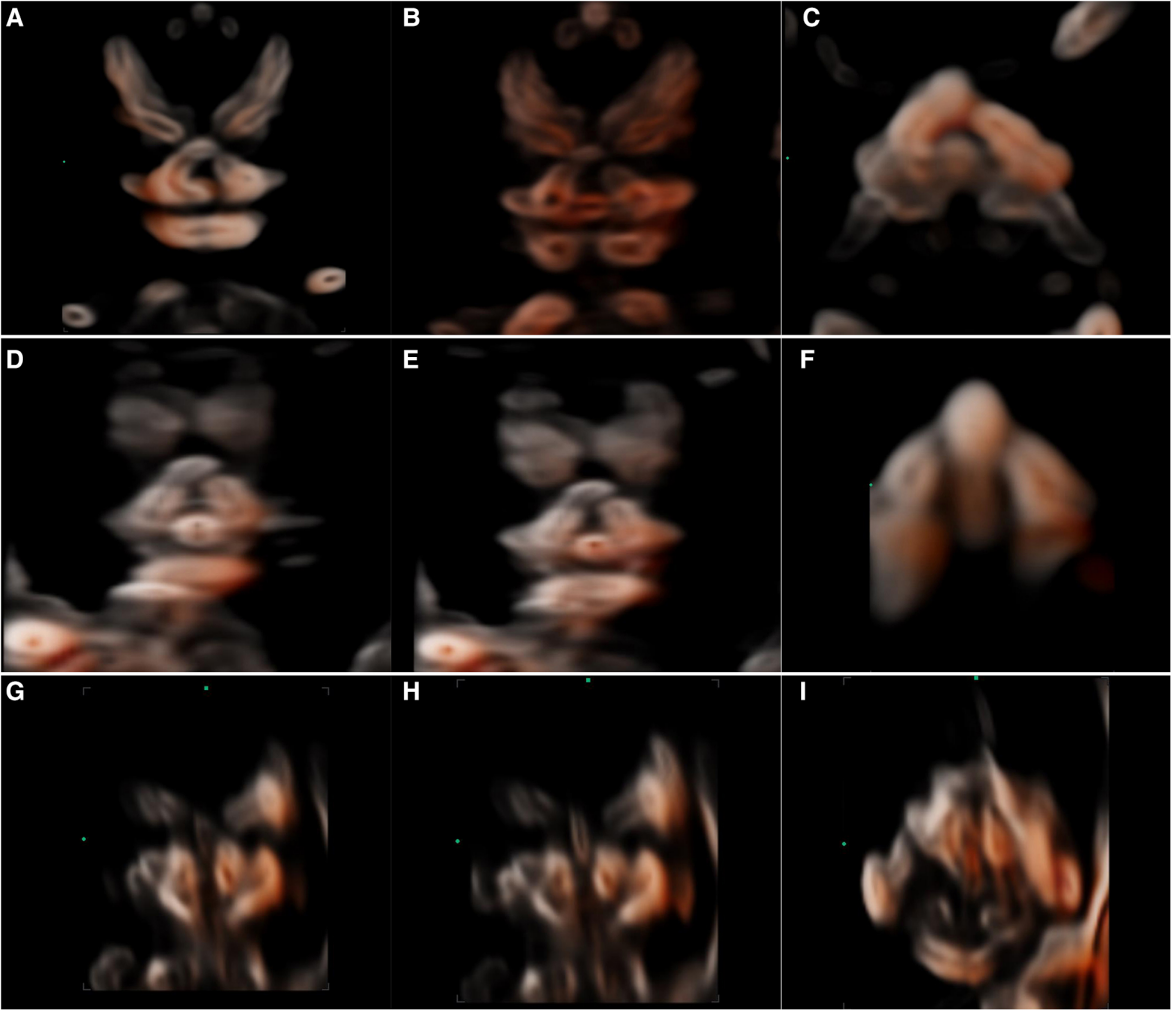
2D and 3D-CRV views of cleft palate. (**A–C**) Unilateral cleft lip and palate, (**D–F**) bilateral cleft lip and palate, and (**G–I**) median cleft lip and palate.

The fetal palatal structure was assessed in 2D view. The 3D data were obtained smoothly, and the collection time was approximately 1.5–2.2 s. Fetal palates were displayed comprehensively by the 3D-CRV technique in all healthy fetuses (Figure 4). Unilateral, bilateral, and median cleft palates with or without lips were visually displayed by the 3D-CRV technique in the cleft palate group (Figure 5).

## 4. Discussion

In our study, there were 23 fetuses with cleft palates with cleft lip and one fetus had cleft palate alone. No fetus had a cleft lip alone in our study. This was also reported in other studies, in which cleft lip and palate were diagnosed in the first trimester (26). The lip is thinner in the first trimester than in the second-third trimester. It is easy to misdiagnose cleft lip in the first trimester. Furthermore, cleft lip alone has the most favorable prognosis. Berge et al. found that fetuses with isolated cleft lips had no aneuploidy (27). Therefore, we speculate that it is more meaningful to examine the palate than the lip in the first trimester.

In our study, the fetuses in the cleft palate group had a high rate of other concomitant structural or chromosomal abnormalities and a relatively poor prognosis. The higher intrauterine mortality may be related to the combination of multiple malformations. In our study, 16 fetuses with cleft palate suffered intrauterine death in the late first trimester and the early second trimester. These fetuses are not included in previous studies on fetal cleft lip and palate in the second–third trimester. This may be one of the reasons for the high rate of other concomitant structural or chromosomal abnormalities and poor prognosis in our study. The findings from the study of Wu et al. were consistent with ours (26).

The coronal view of RNT and the midsagittal view was obtained easily in all fetuses. This may be related to the lack of ossification of all facial structures surrounding the palate, which avoids shadowing from bones and favors visualization (24). This was also confirmed by the study of Martinez-Ten et al. (17). In view of RNT, the frontal processes of the maxilla are at both sides, and the base is the primary palate. The primary cleft palate should be considered when detecting absence at the base of RNT. The vomeromaxillary junction can be seen in the midsagittal view. The “superimposedline” sign is used to assess whether the secondary palate is continuous. In the coronal view of RNT combined with the midsagittal view, the fetal palate is visualized better in the first trimester.

From the 3D datasets using multiplanar mode and CRV imaging, a series of sagittal, coronal, and axial views were obtained. The palate was assessed comprehensively by a series of coronal CRV views in our study. The palate can be visualized as a whole in a transverse CRV view. With those views, the cleft palate in our study was all visualized intuitively and classified. In the cleft palate group, 15 of 24 (62.5%) fetuses (12/15 bilateral, 2/2 median, and 1/1 cleft palate alone) showed other anatomical or chromosomal abnormalities. Unilateral cleft palate with lip was not associated with other anatomical or chromosomal abnormalities. Therefore, in the diagnosis and classification of cleft palate in the first trimester, the 3D-CRV technique can be used as an important auxiliary tool for 2D ultrasonography.

In this study, 3D-CRV seems to be particularly accurate in the imaging of the fetal palate by using novel contrast enhancement. The CRV technique has been proven to be potentially effective in displaying bony structures (18, 20). In a descriptive imaging study, Lees et al. used a novel technique to qualitatively evaluate the performance in healthy fetuses and those with cleft lip alone or cleft palate and lip in the second trimester (23). Their results showed that the cleft of the alveolar ridge and the palate was imaged at a higher definition with Crystal Vue compared with conventional multiplanar mode.

The 3D-CRV technique is feasible to observe the fetal palate and diagnose cleft palate with or without cleft lip in the first trimester without additional scanning time. All 3D data were well obtained only once, and the collection time was approximately 1.5–2.2 s in our study. The main disadvantages of 3D crystal imaging are similar to those of other 3D modes. For example, when obtaining 3D volume, fetal movement may significantly affect the quality of images. If the 2D images are not clear, it may also affect the quality of reconstructed 3D images.

## 5. Conclusion

It is feasible to identify the palate by 3D-CRV in the first trimester in both healthy and cleft palate fetuses. Together with 2D ultrasonography as a complementary diagnostic tool, 3D-CRV is helpful in classifying the cleft palate with a reasonable degree of certainty. When a cleft palate is suspected on 2D ultrasonography, further 3D scanning is recommended. Nevertheless, our results are unlikely to be generalized to the low-risk population, as this study was carried out in a highly selected and nonrepresentative population. More studies with large sample sizes are required to confirm our findings.

## Data Availability

The original contributions presented in the study are included in the article; further inquiries can be directed to the corresponding author.
